# *N*-Cyano-7α-methoxy­carbonyl-6,14-*endo*-ethenotetra­hydro­northebaine

**DOI:** 10.1107/S1600536809032450

**Published:** 2009-08-22

**Authors:** Mustafa Odabaşoğlu, Serkan Yavuz, Özgür Pamir, Orhan Büyükgüngör, Yılmaz Yıldırır

**Affiliations:** a Chemistry Program, Denizli Higher Vocational School, Pamukkale University, TR-20159 Kınıklı, Denizli, Turkey; b Department of Chemistry, Faculty of Arts & Science, Gazi University, Ankara, Turkey; c Department of Physics, Faculty of Arts & Science, Ondokuz Mayıs University, TR-55139 Kurupelit Samsun, Turkey

## Abstract

In the title compound (systematic name: methyl 17-cyano-3,6-dimeth­oxy-4,5α-ep­oxy-6,14-*endo*-ethenomorphinan-7-carboxyl­ate), C_23_H_24_N_2_O_5_, the dihydro­furan ring adopts a twist conformation, while the piperidine ring is in a chair conformation. The benzene-fused cyclo­hexene ring adopts an envelope conformation. An intra­molecular C—H⋯O hydrogen bond is observed. Inter­molecular C—H⋯N and C—H⋯O hydrogen bonds form *C*(5) chains along the *a* and *b* axes, respectively, and together they form a three-dimensional network.

## Related literature

For general background, see: Parrish *et al.*(2004 ▶); Bentley & Hardy (1967 ▶); Marton *et al.* (1995 ▶); Derrick *et al.* (2000 ▶); Lenz *et al.* (1986 ▶); Hoskin & Hanks (1991 ▶); Takemori *et al.* (1972 ▶); Liu *et al.* (2005 ▶). For the synthesis, see: Odabaşoğlu *et al.* (2009 ▶). For graph-set notation, see: Bernstein *et al.* (1995 ▶); Etter (1990 ▶). For ring conformations, see: Cremer & Pople (1975 ▶).
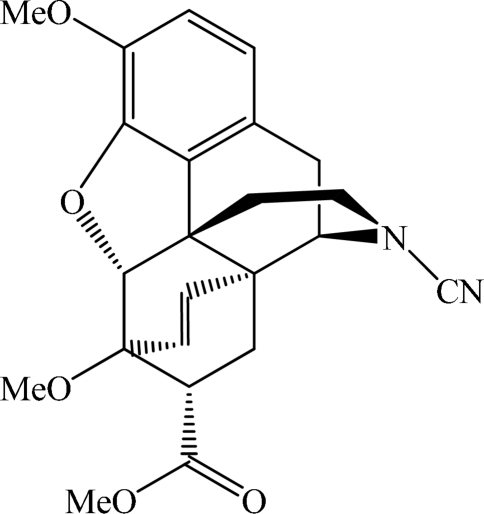


## Experimental

### 

#### Crystal data

C_23_H_24_N_2_O_5_
*M* *_r_* = 408.44
Orthorhombic, 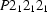

*a* = 7.1880 (3) Å
*b* = 11.1380 (4) Å
*c* = 24.6389 (10) Å
*V* = 1972.59 (13) Å^3^
*Z* = 4
Mo *K*α radiation
μ = 0.10 mm^−1^
*T* = 296 K
0.63 × 0.44 × 0.27 mm

#### Data collection

Stoe IPDS 2 diffractometer
Absorption correction: integration (*X-RED32*; Stoe & Cie, 2002 ▶) *T*_min_ = 0.953, *T*_max_ = 0.975
9908 measured reflections
2328 independent reflections
2035 reflections with *I* > 2σ(*I*)
*R*_int_ = 0.021

#### Refinement

*R*[*F* ^2^ > 2σ(*F* ^2^)] = 0.036
*wR*(*F* ^2^) = 0.089
*S* = 1.04
2328 reflections
274 parameters
H-atom parameters constrained
Δρ_max_ = 0.20 e Å^−3^
Δρ_min_ = −0.20 e Å^−3^

### 

Data collection: *X-AREA* (Stoe & Cie, 2002 ▶); cell refinement: *X-AREA*; data reduction: *X-RED32* (Stoe & Cie, 2002 ▶); program(s) used to solve structure: *SHELXS97* (Sheldrick, 2008 ▶); program(s) used to refine structure: *SHELXL97* (Sheldrick, 2008 ▶); molecular graphics: *ORTEP-3 for Windows* (Farrugia, 1997 ▶); software used to prepare material for publication: *WinGX* (Farrugia, 1999 ▶).

## Supplementary Material

Crystal structure: contains datablocks I, global. DOI: 10.1107/S1600536809032450/ci2873sup1.cif

Structure factors: contains datablocks I. DOI: 10.1107/S1600536809032450/ci2873Isup2.hkl

Additional supplementary materials:  crystallographic information; 3D view; checkCIF report

## Figures and Tables

**Table 1:** Hydrogen-bond geometry (Å, °)

*D*—H⋯*A*	*D*—H	H⋯*A*	*D*⋯*A*	*D*—H⋯*A*
C20—H20*A*⋯O5	0.96	2.55	3.120 (3)	118
C3—H3⋯O1^i^	0.93	2.54	3.399 (3)	153
C9—H9⋯N2^ii^	0.98	2.49	3.467 (4)	179

Symmetry codes: (i) 
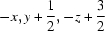
; (ii) 
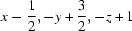
.

**Fig. 1.**
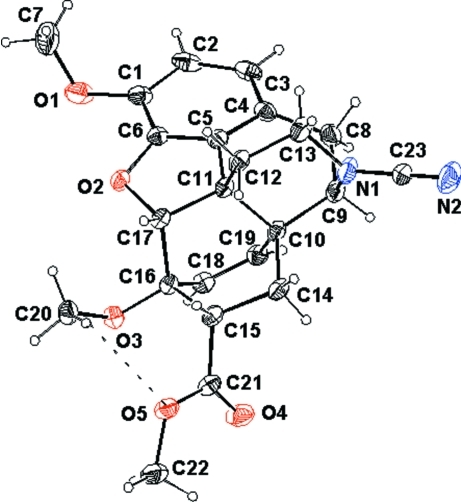
A view of (I), with the atomic numbering scheme. Displacement ellipsoids are drawn at the 15% probability level. The dashed line indicates a hydrogen bond.

**Fig. 2.**
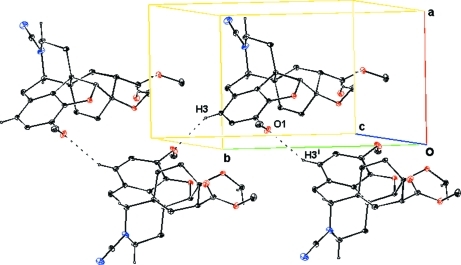
Part of the crystal structure of (I), showing the formation of a C(5) chain along the b axis. H atoms not involved in hydrogen bonds (dashed lines) have been omitted for clarity [symmetry code: (i) -x, 1/2 + y, 3/2 - z].

**Fig. 3.**
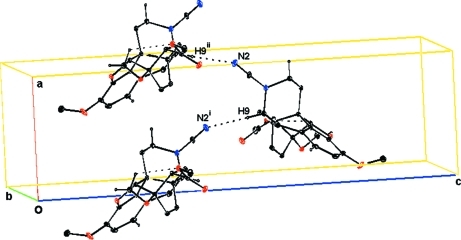
Part of the crystal structure of (I), showing the formation of a C(5) chain along the a axis. H atoms not involved in hydrogen bonds (dashed lines) have been omitted for clarity [symmetry codes: (i) x - 1/2, 3/2 - y, 1 - z; (ii) x + 1/2, 3/2 - y, 1 - z].

**Fig. 4.**
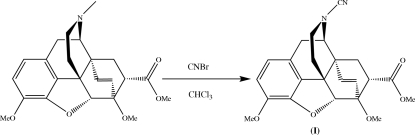
Preparation of the title compound.

## supplementary crystallographic information

### Comment

Morphine alkaloids and semisynthetic derivatives are important drugs for the relief of severe pain. A wide variety of modifications of the well known alkaloids thebaine, codeine and morphine have been described. The Diels-Alder adducts of the thebaine are key intermediates in the synthesis of the potent opioid analgesics (Parrish *et al.*, 2004). Diels-Alder reactions between thebaine and dienophiles predominantly give rise to 7α adducts, and the corresponding 7β adducts have received little attention due to their difficulty of preparation (Bentley & Hardy 1967; Marton *et al.*, 1995; Derrick *et al.*,  2000).

The nature of the substituent at the nitrogen atom in morphine alkaloids is a significant factor having both quantitative and qualitative influence on their pharmacological activity (Lenz *et al.*, 1986). The synthesis and pharmacological activities of 6,14-endoethanomorphinan derivatives have been extensively studied. The typical examples of the pharmacological active compounds were reported in the literature such as buprenorphine (Hoskin & Hanks 1991), etorphine (Takemori *et al.*, 1972) and thienorphine (Liu *et al.*, 2005).

The overall view and atom-labelling of the molecule of (I) are displayed in Fig. 1. The five-membered ring [O2/C6/C5/C11/C17] adopts a twist conformation. Rings A (N1/C9/C10/C11/C12/C13), B(C4/C5/C11/C10/C9/C8), C(C10/C11/C17/C16/C18/C19) and D(C10/C14/C15/C16/C18/C19) are not planar, having total puckering amplitudes, Q_T_, of 0.596 (3) Å, 0.582 (2) Å, 0.768 (2)Å and 0.823 (2) Å, respectively. Rings A, B, C and D adopt chair, envelope, distorted boat and distorted boat conformations, respectively [for ring A: φ = 96 (2)° and θ = 9.4 (3)°; for ring B: φ = 350.1 (3)° and θ = 125.3 (3)°; for ring C: φ = 179.0 (2)° and θ = 90.8 (2)°; for ring D: φ = 6.2 (2)° and θ = 85.8 (1)°; Cremer & Pople, 1975). An intramolecular C20—H20A···O5 hydrogen bond is observed (Fig. 1).

The crystal packing is stabilized by intermolecular C9—H9···N2 and C3—H3···O1 hydrogen bonds (Table 1). As shown in Fig. 2 and Fig. 3, each of these hydrogen bonds forms a C(5) chain (Bernstein *et al.*, 1995; Etter, 1990) and together they form a three-dimensional network.

### Experimental

6,14-*endo*-Etheno-7α-methoxycarbonyltetrahydrothebaine was prepared according to the literature method (Odabaşoğlu *et al.*, 2009). For the preparation of the title compound, 6,14-*endo*-etheno-7α-methoxycarbonyltetrahydrothebaine (240 mg, 0,6 mmol) was heated under reflux with cyanogen bromide (85*m* g, 0,8 mmol) in dry chloroform (20 ml) for 24 h and monitored by TLC. After evaporation of the solvent, the reaction mixture was separated by column chromatography, using a mixture of methanol-chloroform (1:1) as the eluant. The *N*-cyanonor compound was recrystallized from methanol in 2 d (m.p.440–441 K).

### Refinement

H atoms were positioned geometrically (C-H = 0.93–0.98 Å) and refined using a riding model with *U*_iso_(H) = 1.2*U*_eq_(C) and 1.5*U*_eq_(methyl C). A rotating–group model was used for the methyl groups. In the absence of significant anomalous scattering, 1694 Friedel pairs were merged in the final refinement.

### Crystal data

**Table:**

C_23_H_24_N_2_O_5_	*F*(000) = 864
*M**_r_* = 408.44	*D*_x_ = 1.375 Mg m^−^^3^
Orthorhombic, *P*2_1_2_1_2_1_	Mo *K*α radiation, λ = 0.71073 Å
Hall symbol: P 2ac 2ab	Cell parameters from 9908 reflections
*a* = 7.1880 (3) Å	θ = 1.8–28.0°
*b* = 11.1380 (4) Å	µ = 0.10 mm^−^^1^
*c* = 24.6389 (10) Å	*T* = 296 K
*V* = 1972.59 (13) Å^3^	Prism, colourless
*Z* = 4	0.63 × 0.44 × 0.27 mm

### Data collection

**Table:**

Stoe IPDS 2 diffractometer	2328 independent reflections
Radiation source: sealed X-ray tube, 12 x 0.4 mm long-fine focus	2035 reflections with *I* > 2σ(*I*)
plane graphite	*R*_int_ = 0.021
Detector resolution: 6.67 pixels mm^-1^	θ_max_ = 26.5°, θ_min_ = 2.0°
ω–scan rotation method	*h* = −9→9
Absorption correction: integration (*X-RED32*; Stoe & Cie, 2002)	*k* = −13→13
*T*_min_ = 0.953, *T*_max_ = 0.975	*l* = −30→20
9908 measured reflections	

### Refinement

**Table:**

Refinement on *F*^2^	Primary atom site location: structure-invariant direct methods
Least-squares matrix: full	Secondary atom site location: difference Fourier map
*R*[*F*^2^ > 2σ(*F*^2^)] = 0.036	Hydrogen site location: inferred from neighbouring sites
*wR*(*F*^2^) = 0.089	H-atom parameters constrained
*S* = 1.04	*w* = 1/[σ^2^(*F*_o_^2^) + (0.0504*P*)^2^ + 0.208*P*] where *P* = (*F*_o_^2^ + 2*F*_c_^2^)/3
2328 reflections	(Δ/σ)_max_ = 0.001
274 parameters	Δρ_max_ = 0.20 e Å^−^^3^
0 restraints	Δρ_min_ = −0.20 e Å^−^^3^

### Special details

**Table:**

Geometry. All e.s.d.'s (except the e.s.d. in the dihedral angle between two l.s. planes) are estimated using the full covariance matrix. The cell e.s.d.'s are taken into account individually in the estimation of e.s.d.'s in distances, angles and torsion angles; correlations between e.s.d.'s in cell parameters are only used when they are defined by crystal symmetry. An approximate (isotropic) treatment of cell e.s.d.'s is used for estimating e.s.d.'s involving l.s. planes.
Refinement. Refinement of *F*^2^ against ALL reflections. The weighted *R*-factor *wR* and goodness of fit *S* are based on *F*^2^, conventional *R*-factors *R* are based on *F*, with *F* set to zero for negative *F*^2^. The threshold expression of *F*^2^ > σ(*F*^2^) is used only for calculating *R*-factors(gt) *etc*. and is not relevant to the choice of reflections for refinement. *R*-factors based on *F*^2^ are statistically about twice as large as those based on *F*, and *R*- factors based on ALL data will be even larger.

### Fractional atomic coordinates and isotropic or equivalent isotropic displacement parameters (Å^2^)

**Table:**

	*x*	*y*	*z*	*U*_iso_*/*U*_eq_	
C1	0.1582 (3)	0.5657 (2)	0.76990 (11)	0.0558 (6)	
C2	0.1229 (3)	0.6871 (2)	0.76212 (13)	0.0651 (8)	
H2	0.0430	0.7264	0.7859	0.078*	
C3	0.2027 (3)	0.7513 (2)	0.72016 (12)	0.0584 (7)	
H3	0.1697	0.8312	0.7148	0.070*	
C4	0.3316 (3)	0.6975 (2)	0.68598 (10)	0.0494 (5)	
C5	0.3793 (3)	0.5805 (2)	0.69774 (9)	0.0431 (5)	
C6	0.2847 (3)	0.5129 (2)	0.73515 (9)	0.0438 (5)	
C7	0.0990 (5)	0.5179 (3)	0.86177 (15)	0.0929 (11)	
H7A	0.2283	0.5034	0.8690	0.139*	
H7B	0.0245	0.4650	0.8836	0.139*	
H7C	0.0693	0.5997	0.8705	0.139*	
C8	0.3985 (4)	0.7488 (2)	0.63180 (11)	0.0619 (7)	
H8A	0.4784	0.8171	0.6391	0.074*	
H8B	0.2912	0.7783	0.6120	0.074*	
C9	0.5055 (4)	0.6599 (2)	0.59495 (10)	0.0550 (6)	
H9	0.4781	0.6798	0.5570	0.066*	
C10	0.4513 (3)	0.5280 (2)	0.60475 (9)	0.0445 (5)	
C11	0.5026 (3)	0.5014 (2)	0.66483 (8)	0.0405 (4)	
C12	0.7122 (3)	0.5215 (2)	0.67525 (9)	0.0492 (5)	
H12A	0.7375	0.5101	0.7136	0.059*	
H12B	0.7827	0.4618	0.6553	0.059*	
C13	0.7765 (4)	0.6444 (3)	0.65865 (10)	0.0615 (7)	
H13A	0.7284	0.7040	0.6837	0.074*	
H13B	0.9113	0.6482	0.6594	0.074*	
C14	0.5448 (4)	0.4336 (2)	0.56775 (9)	0.0530 (6)	
H14A	0.4972	0.4407	0.5310	0.064*	
H14B	0.6782	0.4466	0.5668	0.064*	
C15	0.5020 (3)	0.3077 (2)	0.59042 (9)	0.0464 (5)	
H15	0.6147	0.2777	0.6083	0.056*	
C16	0.3421 (3)	0.31523 (19)	0.63470 (9)	0.0414 (5)	
C17	0.4396 (3)	0.37595 (19)	0.68281 (8)	0.0413 (5)	
H17	0.5488	0.3285	0.6932	0.050*	
C18	0.1924 (3)	0.3955 (2)	0.61281 (9)	0.0460 (5)	
H18	0.0696	0.3701	0.6096	0.055*	
C19	0.2453 (3)	0.5040 (2)	0.59850 (9)	0.0476 (5)	
H19	0.1625	0.5615	0.5856	0.057*	
C20	0.3673 (4)	0.1229 (2)	0.67858 (11)	0.0580 (6)	
H20A	0.4882	0.1129	0.6627	0.087*	
H20B	0.3058	0.0465	0.6803	0.087*	
H20C	0.3802	0.1550	0.7145	0.087*	
C21	0.4457 (3)	0.2176 (2)	0.54757 (10)	0.0524 (6)	
C22	0.4577 (6)	0.0119 (3)	0.52491 (12)	0.0800 (9)	
H22A	0.3286	−0.0057	0.5309	0.120*	
H22B	0.5310	−0.0578	0.5331	0.120*	
H22C	0.4760	0.0340	0.4876	0.120*	
C23	0.8136 (4)	0.7260 (2)	0.56914 (10)	0.0583 (6)	
N1	0.7083 (4)	0.6693 (2)	0.60326 (9)	0.0710 (7)	
N2	0.9105 (5)	0.7735 (3)	0.53969 (12)	0.1007 (10)	
O1	0.0625 (3)	0.4968 (2)	0.80685 (10)	0.0924 (8)	
O2	0.3187 (2)	0.39121 (14)	0.73017 (6)	0.0487 (4)	
O3	0.2608 (2)	0.20286 (14)	0.64642 (7)	0.0496 (4)	
O4	0.3500 (3)	0.2385 (2)	0.50871 (8)	0.0753 (6)	
O5	0.5139 (3)	0.10971 (16)	0.55952 (7)	0.0611 (5)	

### Atomic displacement parameters (Å^2^)

**Table:**

	*U*^11^	*U*^22^	*U*^33^	*U*^12^	*U*^13^	*U*^23^
C1	0.0440 (11)	0.0639 (15)	0.0594 (15)	−0.0158 (11)	0.0118 (11)	−0.0196 (12)
C2	0.0411 (12)	0.0655 (17)	0.089 (2)	−0.0039 (12)	0.0063 (13)	−0.0372 (15)
C3	0.0479 (13)	0.0478 (13)	0.0794 (18)	0.0029 (11)	−0.0166 (13)	−0.0162 (12)
C4	0.0462 (12)	0.0453 (12)	0.0565 (14)	−0.0042 (10)	−0.0137 (11)	−0.0037 (10)
C5	0.0380 (10)	0.0512 (12)	0.0400 (11)	−0.0009 (9)	−0.0052 (9)	−0.0007 (9)
C6	0.0376 (10)	0.0510 (12)	0.0429 (11)	−0.0012 (9)	−0.0001 (9)	−0.0062 (9)
C7	0.076 (2)	0.110 (3)	0.093 (3)	−0.015 (2)	−0.0151 (18)	0.037 (2)
C8	0.0750 (17)	0.0485 (13)	0.0622 (16)	−0.0068 (13)	−0.0176 (14)	0.0088 (12)
C9	0.0606 (15)	0.0632 (15)	0.0413 (12)	−0.0127 (12)	−0.0101 (11)	0.0129 (10)
C10	0.0458 (11)	0.0529 (12)	0.0347 (10)	−0.0025 (10)	−0.0048 (9)	0.0069 (9)
C11	0.0374 (10)	0.0496 (11)	0.0346 (10)	0.0006 (9)	−0.0008 (8)	0.0036 (9)
C12	0.0375 (11)	0.0722 (15)	0.0379 (11)	−0.0018 (10)	−0.0014 (9)	0.0052 (11)
C13	0.0485 (13)	0.0891 (19)	0.0469 (14)	−0.0188 (13)	−0.0052 (11)	0.0121 (12)
C14	0.0539 (13)	0.0704 (15)	0.0348 (11)	−0.0025 (12)	0.0042 (10)	0.0017 (10)
C15	0.0399 (11)	0.0609 (13)	0.0384 (11)	0.0064 (10)	0.0002 (9)	−0.0008 (10)
C16	0.0379 (10)	0.0469 (11)	0.0395 (11)	0.0033 (9)	0.0016 (9)	0.0026 (9)
C17	0.0388 (10)	0.0502 (11)	0.0348 (11)	0.0079 (9)	0.0018 (9)	0.0037 (9)
C18	0.0358 (10)	0.0554 (13)	0.0467 (12)	0.0034 (10)	−0.0052 (9)	−0.0014 (10)
C19	0.0465 (11)	0.0509 (12)	0.0454 (11)	0.0058 (10)	−0.0129 (10)	0.0016 (10)
C20	0.0634 (15)	0.0523 (13)	0.0582 (15)	0.0050 (12)	0.0007 (12)	0.0119 (12)
C21	0.0518 (13)	0.0645 (15)	0.0408 (12)	0.0066 (12)	0.0021 (10)	−0.0039 (11)
C22	0.117 (3)	0.0672 (17)	0.0557 (16)	0.0099 (19)	0.0001 (17)	−0.0166 (14)
C23	0.0749 (16)	0.0556 (14)	0.0443 (13)	−0.0099 (13)	0.0118 (12)	−0.0001 (11)
N1	0.0676 (14)	0.0989 (17)	0.0467 (12)	−0.0295 (13)	−0.0056 (10)	0.0215 (12)
N2	0.101 (2)	0.135 (3)	0.0660 (18)	−0.026 (2)	0.0247 (16)	0.0189 (17)
O1	0.0946 (15)	0.0935 (15)	0.0891 (16)	−0.0483 (13)	0.0573 (13)	−0.0418 (13)
O2	0.0554 (8)	0.0505 (9)	0.0403 (8)	0.0008 (7)	0.0127 (7)	0.0014 (7)
O3	0.0454 (8)	0.0485 (8)	0.0548 (9)	0.0022 (7)	0.0009 (7)	0.0038 (7)
O4	0.0877 (14)	0.0788 (12)	0.0596 (12)	0.0128 (12)	−0.0285 (11)	−0.0099 (9)
O5	0.0748 (11)	0.0627 (10)	0.0459 (9)	0.0161 (10)	−0.0023 (9)	−0.0078 (8)

### Geometric parameters (Å, °)

**Table:**

C1—O1	1.376 (3)	C13—N1	1.476 (3)
C1—C6	1.381 (3)	C13—H13A	0.97
C1—C2	1.389 (4)	C13—H13B	0.97
C2—C3	1.382 (4)	C14—C15	1.541 (3)
C2—H2	0.93	C14—H14A	0.97
C3—C4	1.388 (4)	C14—H14B	0.97
C3—H3	0.93	C15—C21	1.512 (3)
C4—C5	1.379 (3)	C15—C16	1.587 (3)
C4—C8	1.530 (4)	C15—H15	0.98
C5—C6	1.371 (3)	C16—O3	1.411 (3)
C5—C11	1.489 (3)	C16—C18	1.500 (3)
C6—O2	1.383 (3)	C16—C17	1.534 (3)
C7—O1	1.398 (4)	C17—O2	1.465 (2)
C7—H7A	0.96	C17—H17	0.98
C7—H7B	0.96	C18—C19	1.315 (3)
C7—H7C	0.96	C18—H18	0.93
C8—C9	1.548 (4)	C19—H19	0.93
C8—H8A	0.97	C20—O3	1.417 (3)
C8—H8B	0.97	C20—H20A	0.96
C9—N1	1.476 (4)	C20—H20B	0.96
C9—C10	1.539 (3)	C20—H20C	0.96
C9—H9	0.98	C21—O4	1.202 (3)
C10—C19	1.513 (3)	C21—O5	1.330 (3)
C10—C14	1.546 (3)	C22—O5	1.441 (3)
C10—C11	1.554 (3)	C22—H22A	0.96
C11—C17	1.534 (3)	C22—H22B	0.96
C11—C12	1.545 (3)	C22—H22C	0.96
C12—C13	1.502 (4)	C23—N2	1.137 (3)
C12—H12A	0.97	C23—N1	1.296 (3)
C12—H12B	0.97		
			
O1—C1—C6	120.1 (2)	N1—C13—H13B	110.0
O1—C1—C2	122.9 (2)	C12—C13—H13B	110.0
C6—C1—C2	116.7 (2)	H13A—C13—H13B	108.3
C3—C2—C1	122.1 (2)	C15—C14—C10	108.61 (18)
C3—C2—H2	119.0	C15—C14—H14A	110.0
C1—C2—H2	119.0	C10—C14—H14A	110.0
C2—C3—C4	120.5 (2)	C15—C14—H14B	110.0
C2—C3—H3	119.8	C10—C14—H14B	110.0
C4—C3—H3	119.7	H14A—C14—H14B	108.3
C5—C4—C3	116.5 (2)	C21—C15—C14	113.87 (19)
C5—C4—C8	117.3 (2)	C21—C15—C16	108.75 (19)
C3—C4—C8	125.3 (2)	C14—C15—C16	110.19 (18)
C6—C5—C4	122.5 (2)	C21—C15—H15	107.9
C6—C5—C11	109.66 (19)	C14—C15—H15	107.9
C4—C5—C11	126.3 (2)	C16—C15—H15	107.9
C5—C6—C1	120.6 (2)	O3—C16—C18	107.77 (17)
C5—C6—O2	113.00 (18)	O3—C16—C17	114.96 (17)
C1—C6—O2	126.1 (2)	C18—C16—C17	110.04 (17)
O1—C7—H7A	109.5	O3—C16—C15	113.17 (17)
O1—C7—H7B	109.5	C18—C16—C15	107.72 (18)
H7A—C7—H7B	109.5	C17—C16—C15	102.94 (16)
O1—C7—H7C	109.5	O2—C17—C16	113.33 (17)
H7A—C7—H7C	109.5	O2—C17—C11	107.43 (16)
H7B—C7—H7C	109.5	C16—C17—C11	108.24 (16)
C4—C8—C9	115.4 (2)	O2—C17—H17	109.3
C4—C8—H8A	108.4	C16—C17—H17	109.3
C9—C8—H8A	108.4	C11—C17—H17	109.3
C4—C8—H8B	108.4	C19—C18—C16	115.9 (2)
C9—C8—H8B	108.4	C19—C18—H18	122.0
H8A—C8—H8B	107.5	C16—C18—H18	122.0
N1—C9—C10	107.2 (2)	C18—C19—C10	114.7 (2)
N1—C9—C8	111.4 (2)	C18—C19—H19	122.6
C10—C9—C8	113.1 (2)	C10—C19—H19	122.6
N1—C9—H9	108.3	O3—C20—H20A	109.5
C10—C9—H9	108.3	O3—C20—H20B	109.5
C8—C9—H9	108.3	H20A—C20—H20B	109.5
C19—C10—C9	113.6 (2)	O3—C20—H20C	109.5
C19—C10—C14	104.20 (19)	H20A—C20—H20C	109.5
C9—C10—C14	116.56 (19)	H20B—C20—H20C	109.5
C19—C10—C11	107.17 (18)	O4—C21—O5	124.2 (2)
C9—C10—C11	105.77 (18)	O4—C21—C15	125.5 (2)
C14—C10—C11	109.19 (18)	O5—C21—C15	110.3 (2)
C5—C11—C17	101.88 (17)	O5—C22—H22A	109.5
C5—C11—C12	113.82 (19)	O5—C22—H22B	109.5
C17—C11—C12	111.83 (18)	H22A—C22—H22B	109.5
C5—C11—C10	105.35 (18)	O5—C22—H22C	109.5
C17—C11—C10	112.28 (18)	H22A—C22—H22C	109.5
C12—C11—C10	111.22 (18)	H22B—C22—H22C	109.5
C13—C12—C11	112.8 (2)	N2—C23—N1	177.9 (3)
C13—C12—H12A	109.0	C23—N1—C9	121.4 (2)
C11—C12—H12A	109.0	C23—N1—C13	119.8 (2)
C13—C12—H12B	109.0	C9—N1—C13	116.3 (2)
C11—C12—H12B	109.0	C1—O1—C7	116.9 (2)
H12A—C12—H12B	107.8	C6—O2—C17	106.83 (16)
N1—C13—C12	108.7 (2)	C16—O3—C20	116.63 (18)
N1—C13—H13A	110.0	C21—O5—C22	116.6 (2)
C12—C13—H13A	110.0		
			
O1—C1—C2—C3	−171.3 (3)	C10—C14—C15—C16	13.0 (2)
C6—C1—C2—C3	3.1 (4)	C21—C15—C16—O3	38.3 (2)
C1—C2—C3—C4	−4.1 (4)	C14—C15—C16—O3	163.76 (18)
C2—C3—C4—C5	−2.6 (3)	C21—C15—C16—C18	−80.7 (2)
C2—C3—C4—C8	166.4 (2)	C14—C15—C16—C18	44.7 (2)
C3—C4—C5—C6	10.5 (3)	C21—C15—C16—C17	162.99 (18)
C8—C4—C5—C6	−159.4 (2)	C14—C15—C16—C17	−71.5 (2)
C3—C4—C5—C11	175.0 (2)	O3—C16—C17—O2	−55.4 (2)
C8—C4—C5—C11	5.1 (3)	C18—C16—C17—O2	66.5 (2)
C4—C5—C6—C1	−11.9 (3)	C15—C16—C17—O2	−178.91 (16)
C11—C5—C6—C1	−178.7 (2)	O3—C16—C17—C11	−174.44 (16)
C4—C5—C6—O2	162.9 (2)	C18—C16—C17—C11	−52.6 (2)
C11—C5—C6—O2	−3.9 (2)	C15—C16—C17—C11	62.0 (2)
O1—C1—C6—C5	179.3 (2)	C5—C11—C17—O2	−10.9 (2)
C2—C1—C6—C5	4.7 (3)	C12—C11—C17—O2	111.03 (19)
O1—C1—C6—O2	5.2 (4)	C10—C11—C17—O2	−123.14 (18)
C2—C1—C6—O2	−169.4 (2)	C5—C11—C17—C16	111.83 (18)
C5—C4—C8—C9	2.8 (3)	C12—C11—C17—C16	−126.25 (19)
C3—C4—C8—C9	−166.1 (2)	C10—C11—C17—C16	−0.4 (2)
C4—C8—C9—N1	−94.0 (3)	O3—C16—C18—C19	−178.53 (19)
C4—C8—C9—C10	26.8 (3)	C17—C16—C18—C19	55.4 (3)
N1—C9—C10—C19	179.2 (2)	C15—C16—C18—C19	−56.1 (3)
C8—C9—C10—C19	56.0 (3)	C16—C18—C19—C10	2.1 (3)
N1—C9—C10—C14	−59.6 (3)	C9—C10—C19—C18	−172.41 (19)
C8—C9—C10—C14	177.2 (2)	C14—C10—C19—C18	59.7 (3)
N1—C9—C10—C11	61.9 (2)	C11—C10—C19—C18	−56.0 (3)
C8—C9—C10—C11	−61.3 (2)	C14—C15—C21—O4	−38.1 (3)
C6—C5—C11—C17	9.0 (2)	C16—C15—C21—O4	85.2 (3)
C4—C5—C11—C17	−157.1 (2)	C14—C15—C21—O5	143.4 (2)
C6—C5—C11—C12	−111.5 (2)	C16—C15—C21—O5	−93.3 (2)
C4—C5—C11—C12	82.3 (3)	N2—C23—N1—C9	−154 (9)
C6—C5—C11—C10	126.37 (19)	N2—C23—N1—C13	45 (9)
C4—C5—C11—C10	−39.8 (3)	C10—C9—N1—C23	133.5 (3)
C19—C10—C11—C5	−57.1 (2)	C8—C9—N1—C23	−102.3 (3)
C9—C10—C11—C5	64.4 (2)	C10—C9—N1—C13	−64.4 (3)
C14—C10—C11—C5	−169.41 (18)	C8—C9—N1—C13	59.9 (3)
C19—C10—C11—C17	53.0 (2)	C12—C13—N1—C23	−141.3 (3)
C9—C10—C11—C17	174.49 (18)	C12—C13—N1—C9	56.2 (3)
C14—C10—C11—C17	−59.3 (2)	C6—C1—O1—C7	114.6 (3)
C19—C10—C11—C12	179.1 (2)	C2—C1—O1—C7	−71.2 (4)
C9—C10—C11—C12	−59.3 (2)	C5—C6—O2—C17	−3.5 (2)
C14—C10—C11—C12	66.8 (2)	C1—C6—O2—C17	170.9 (2)
C5—C11—C12—C13	−64.4 (3)	C16—C17—O2—C6	−110.32 (19)
C17—C11—C12—C13	−179.20 (18)	C11—C17—O2—C6	9.2 (2)
C10—C11—C12—C13	54.4 (3)	C18—C16—O3—C20	−166.3 (2)
C11—C12—C13—N1	−48.9 (3)	C17—C16—O3—C20	−43.2 (2)
C19—C10—C14—C15	−64.4 (2)	C15—C16—O3—C20	74.7 (2)
C9—C10—C14—C15	169.59 (19)	O4—C21—O5—C22	−4.0 (4)
C11—C10—C14—C15	49.9 (2)	C15—C21—O5—C22	174.5 (2)
C10—C14—C15—C21	135.5 (2)		

### Hydrogen-bond geometry (Å, °)

**Table:**

*D*—H···*A*	*D*—H	H···*A*	*D*···*A*	*D*—H···*A*
C20—H20A···O5	0.96	2.55	3.120 (3)	118
C3—H3···O1^i^	0.93	2.54	3.399 (3)	153
C9—H9···N2^ii^	0.98	2.49	3.467 (4)	179

Symmetry codes: (i) −*x*, *y*+1/2, −*z*+3/2; (ii) *x*−1/2, −*y*+3/2, −*z*+1.
